# Evaluation of the Quality and Reliability of YouTube Videos With Turkish Content as an Information Source for Gynecological Cancers During the COVID-19 Pandemic

**DOI:** 10.7759/cureus.44581

**Published:** 2023-09-02

**Authors:** Fırat Ekmez, Murat Ekmez

**Affiliations:** 1 Obstetrics and Gynecology, Private Gynecology and Obstetrics Center, Silopi, Sirnak, TUR; 2 Obstetrics and Gynecology, Haseki Education and Research Hospital, Istanbul, TUR

**Keywords:** gynecologic cancers, covid-19, discern, mici score, youtube videos

## Abstract

Aim

During the COVID-19 pandemic, YouTube became a critical information source for people seeking information about several diseases, including benign and malignant gynecological disorders. This study aimed to evaluate the quality and reliability of YouTube videos with Turkish content as an information source for gynecological cancers during the COVID-19 pandemic.

Methods

The research was performed between December 2nd and 5th, 2020. Two gynecologists searched the terms in Turkish; ‘yumurtalık kanseri, COVID-19’, ‘rahim kanseri, coronavirus’, ‘rahim ağzı kanseri, COVID-19’, ‘kadın üreme sistemi kanseri, coronavirus’, and ‘jinekolojik kanserler, COVID-19’. on YouTube. ‘Yumurtalık kanseri’, ‘rahim kanseri’, ‘rahim agzı kanseri’, ‘kadın üreme sistemi kanserleri’ and ‘jinekolojik kanserler’ are the translations for "ovarian cancer, COVID-19", "endometrial cancer, coronavirus", "cervical cancer, COVID-19", "female reproductive system cancers, coronavirus", and "gynecological cancers, COVID-19" in the Turkish language, respectively. Videos were categorized into three groups depending on the upload source: the informative group, the personal experience group, and the news agency group. Moreover, DISCERN, the Medical Information and Content Index (MICI), the Video Information and Quality Index (VIQI) were evaluated.

Results

A total of 57 videos were categorized as informative. Additionally, 22 videos and 18 videos were classified as patient experience videos and new agency videos, respectively. Patients who experienced videos had a significantly higher view number (p=0.001). The number of dislikes and DISCERN score were markedly better in favor of informative videos (p=0.009 and p=0.001). The MICI score was calculated as 13.0±1.8 for informative videos. The total VIQI score was 11.9 for informative videos, 8.8 for patient experience videos, and 7.2 for new agency videos (p = 0.001).

Conclusions

YouTube videos with Turkish content about gynecological cancers are easily accessible resources during the COVID-19 pandemic. Patient-published videos are the most preferred YouTube videos by Turkish citizens, and informative videos have a considerably lower dislike rate. According to the MICI score and significantly better DISCERN and VIQI scores, informative videos have acceptable quality.

## Introduction

The new coronavirus infection (COVID-19), first detected in December 2019 in China, caused a global crisis. The World Health Organization accepted COVID-19 as a public health emergency of international concern on March 11, 2020 [1]. According to the latest data, over 769 million people were infected, and 13 million individuals died due to COVID-19. Recently, 229 countries around the world have confirmed COVID-19 cases [2]. Due to the easy and quick transmission of COVID-19, many protection rules were applied, including increasing social distancing, restricting collective action, and limiting public transportation. Additionally, professional health providers started to fight COVID-19, and many outpatient polyclinic appointments and elective surgeries were postponed [3]. Due to limitations in access to the health system, many individuals try to get information from other sources, including books, television, or the Internet.

Social media is increasingly preferred as an information source on the grounds that it is easy to access, free of charge, and provides resource diversity. Additionally, Freeman and colleagues claimed that posts with video content were more attractive than audio and written posts [4]. YouTube is the second-largest social media platform with 1.9 billion users, and 5 billion videos are watched on YouTube every single day [5]. Previous studies stated the impact of YouTube videos on getting information about diseases' symptoms, diagnosis, treatment, and follow-up. However, Bora et al. showed the insufficiency and impropriety of YouTube videos about the Zika virus [6]. In another study, Kumar et al. stated that misleading YouTube videos about hypertension had a higher view rate [7].

YouTube has no standardized behavior to analyze shared videos' properties and quality; thus, uploaded videos may contain useful, inadequate, or misleading information. Turkish citizens generally prefer YouTube videos with Turkish content. No study evaluated the use of these videos as an information source for gaining knowledge about gynecological cancers. We thought that the scientific assessment of the informative value of YouTube videos for Turkish citizens would be helpful for physicians dealing with gynecological cancer patients at the time of COVID-19. This study aimed to evaluate the quality and reliability of YouTube videos with Turkish content as an information source for gynecological cancers during the COVID-19 pandemic.

## Materials and methods

In this study, the properties of the videos published on YouTube about cancers of female reproductive organs and watched by the Turkish population during the COVID-19 pandemic were evaluated between December 2 and 5, 2020. The study was conducted in Haseki Education and Research Hospital, Istanbul, Turkey. The authors searched Turkish terms, possibly used by citizens, as the following: ‘yumurtalık kanseri, COVID-19’, ‘rahim kanseri, coronavirus’, ‘rahim ağzı kanseri, COVID-19’, ‘kadın üreme sistemi kanserleri, coronavirus’, and ‘jinekolojik kanserler, COVID-19’. on YouTube. ‘Yumurtalık kanseri’, ‘rahim kanseri’, ‘rahim agzı kanseri’, ‘kadın üreme sistemi kanserleri’ and ‘jinekolojik kanserler' are the translations for "ovarian cancer, COVID-19", "endometrial cancer, coronavirus", "cervical cancer, COVID-19", "female reproductive system cancers, coronavirus", and "gynecological cancers, COVID-19" in the Turkish language, respectively.

The YouTube ranking system demonstrated that the most popular videos are between two to 15 minutes long. Thus, we only analyzed videos with a duration of two minutes to 15 minutes. Also, personal propaganda was excluded from the study. Finally, 144 YouTube videos were analyzed. YouTube videos with different languages (14 videos) and videos with irrelevant subjects (33 videos) were excluded. The playlist was created with the remaining 97 videos, and all videos were evaluated cautiously by two independent gynecologists. Due to not using any patient data in this study, institutional ethics committee approval was not achieved.

The length of the video, duration on YouTube (days), number of views and comments, and number of ‘likes’ and ‘dislikes’ were noted for each video. Videos were classified into three groups according to the uploaders of the videos: ‘health care providers’, ‘new agencies’, and ‘nonprofessional individuals’. Videos were labeled into two groups (patients or healthcare workers) according to the target population. Videos that contained accurate scientific information about COVID-19 epidemiology, symptoms, and course of disease were categorized as informative groups. Videos that included patients’ experiences of COVID-19 were named the personal experience group, and videos with news uploaded by news sources were named the news update group.

The DISCERN score (from zero to five points) was developed to obtain an objective evaluation of the quality of the videos. The scale has five questions: ‘‘Were aims clear and achieved?’, ‘Were the sources of information reliable?’, ‘Is the information balanced and unbiased?’, ‘Are additional resources to learn provided?’, and ‘Does the video address areas of controversy or uncertainty?’’ with answers of yes or no, and each ‘yes’ answer equals one point and shows a positive aspect [8]. Furthermore, the quality of videos was assessed by the Medical Information and Content Index (MICI) and the Video Information and Quality Index (VIQI). The MICI questionnaire (ranging from zero to five points) investigated the presence of five subjects, including disease prevalence, transmission, symptoms, screening tests, and treatment methods. Videos get one point for each mentioned subject [9]. The VIQI (from one point of poor quality to five points of high quality) was created to analyze the quality of information on websites. The VIQI scale included four sub-evaluations, including the flow of information, the accuracy of the information, video quality (one point each for use of still images, use of animation, interviews with individuals in the community, video captions, and use of a report summary), and precision (level of coherence between video title and content) [10].

IBM SPSS Statistics for Windows, Version 20.0 (released 2011; IBM Corp., Armonk, New York, United States) was used. The normality of the distribution of the variables was checked by the Shapiro-Wilk test and Q-Q plots. An ANOVA test was used for comparison of the normally distributed variable between the three groups. The Kruskal-Wallis test was preferred for nonnormally distributed data. A Tukey post-hoc test was used to compute pairwise comparisons. Quantitative data are shown as mean ± standard error values. The data were analyzed at a 95% confidence level, and a P-value of less than 0.05 was accepted as statistically significant.

## Results

**Figure 1:**
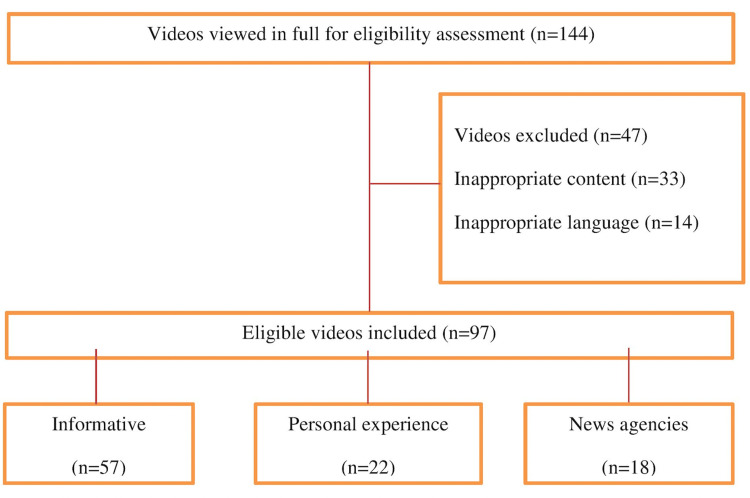
Flowchart of the study

At the end of the evaluation, 97 YouTube videos were included in the present study. Due to the inappropriate content (n = 33) and vague language (n = 14), 47 videos were excluded from the research. According to the video type, 57 videos are categorized as informative videos. Additionally, 22 videos and 18 videos were classified as patient experience videos and new agency videos, respectively (Figure 1).

Patient experience videos had a significantly higher view number than informative and new agency videos (p= 0.001). In contrast, video duration, period on YouTube, views per day, and the number of likes and comments were comparable between groups (p= 0.490, p= 0.419, p= 0.289, p= 0.270, and p= 0.155, respectively). Additionally, the number of dislikes and DISCERN score were significantly higher in favor of informative videos (p= 0.009 and p= 0.001). The majority of informative videos were uploaded by professional healthcare providers (p= 0.001). Almost two-thirds of informative videos targeted professional health workers, but patients' experience videos and new agency videos mostly focused on the public (p= 0.001) (Table 1). A pairwise comparison of three groups revealed that patients' experience videos had a significantly higher view rate (p= 0.006 for informative videos and p= 0.005 for new agency videos), informative videos had a significantly lower dislike rate (p= 0.001 for both patient's experience videos and new agency videos), and a higher DISCERN score (p= 0.001 for both patients experience videos and new agency videos) (Table 2).

**Table 1:** Comparison of video groups’ characteristics

Characteristics	Informative	Patient- published	News agency- published	p-value
Number of videos	57	22	18	
Audience interaction properties (Mean±SD)				
Views number	298.5±75.6	3329.7±1310.6	288.4±72.3	0.001
Video duration (min)	7.4±0.8	6.2±2.1	6.5±1.5	0.490
Period on YouTube (days)	158.7±10.9	164.8±16.9	185.9±45.2	0.419
Views per day	1.9±0.4	15.6±6.4	3.6±1.3	0.289
Likes	12.3±6.4	32.3±13.3	22.9±4.0	0.270
Dislikes	0.5±0.3	2.8±1.2	1.8±1.1	0.009
Comments	2.4±1.1	16.8±7.8	11.7±7.0	0.155
DISCERN score	3.2±1.0	1.6±0.3	0.8±0.4	0.001
Source of upload n(%)				0.001
Professional healthcare providers	42 (73.6%)	-	2 (11.1%)	
Non-professional individuals	11 (19.3%)	10 (45.4%)	-	
News agencies	4 (7.0%)	12 (54.6%)	16 (88.9%)	
Target audience n(%)				0.001
For professional healthcare providers	38 (66.7%)	-	5 (27.8%)	
For public	19 (33.3%)	22 (100%)	13 (72.2%)	

**Table 2:** Pairwise comparisons of video groups according to statistically significant parameters univariate analysis

Characteristics	p-value		
	Informative vs. Patient-published	Informative vs. News agency- published	Patient-published vs. News agency-published
Views number	0.006	0.995	0.005
Dislikes	0.001	0.001	0.779
DISCERN score	0.001	0.001	0.462

Clinical symptoms of COVID-19 and information about transmission properties were the most discussed subjects in informative videos (72.1% and 63.9%, respectively) (Table 3). Moreover, information about COVID-19 prevalence and screening tests was available in 54 and 27 videos, respectively. In 51 videos, treatment and/or treatment outcomes were discussed. The mean MICI score was calculated as 13.0±1.8 for informative videos.

**Table 3:** Analysis of informative videos according to MICI score MICI: Medical Information and Content Index

Component of MICI scale	Videos with information n(%)	MICI score (Mean±SD)
Prevalence	54 (55.6%)	2.9±0.4
Transmission	62 (63.9%)	3.1±0.2
Clinical symptoms	70 (72.1%)	1.9±0.5
Screening/tests	27 (27.8%)	2.3±0.4
Treatment/outcomes	51 (52.6%)	2.8±0.6
Total MICI score		13.0±1.8

Informative videos got significantly better scores on the inflow of information and information accuracy than patient experience videos and new agency videos (2.7 vs. 1.5 vs. 1.3, p= 0.001 and 3.5 vs. 1.4 vs. 1.3, p= 0.001). The quality and precision of the videos for all three groups were similar (p= 0.078 and p= 0.052). The total VIQI score was 11.9 for informative videos, 8.8 for patients' experience videos, and 7.2 for new agency videos (p= 0.001) (Table 4). Pairwise comparisons of video groups according to VIQI scores demonstrated that flow information, information accuracy, and total VIQI score were significantly better in favor of informative videos (Table 5). The kappa coefficient of agreement regarding the DISCERN score, MICI score, and VIQI score was 0.76 (p<0.001), 0.87 (p<0.001), and 0.77 (p<0.001), respectively.

**Table 4:** Detailed content analysis of video groups based on VIQI scores VIQI: Video Information and Quality Index

	Informative (Mean±SD)	Patient-published (Mean±SD)	News agency-published (Mean±SD)	p-value
Flow of information	2.7±0.4	1.5±0.3	1.3±0.4	0.001
Information accuracy	3.5±0.6	1.4±0.6	1.3±0.3	0.001
Quality	2.2±0.2	1.4±0.2	1.7±0.4	0.078
Precision	3.5±0.7	4.5±0.8	2.9±0.8	0.052
Total VIQI score	11.9±1.3	8.8±0.9	7.2±0.9	0.001

**Table 5:** Pairwise comparisons of video groups according to VIQI scores VIQI: Video Information and Quality Index

	p-value		
	Informative vs. Patient- published	Informative vs. News agency- published	Patient- published vs. News agency- published
Flow of information	0.001	0.001	0.486
Information accuracy	0.002	0.001	0.986
Total VIQI score	0.001	0.005	0.246

## Discussion

Easy and rapid access to knowledge on the Internet has changed many habits, including trading, communication, and obtaining health information. Recently, YouTube was preferred by almost 95% of internet users and accepted as the second most prominent social media application [11]. Entering COVID-19 prevention rules into daily life and limitations in accessing a professional health system have prompted people to get health information from social media. Thus, the COVID-19 pandemic gave us an opportunity to analyze the characteristics of YouTube videos about COVID-19 and gynecologic cancers.

The DISCERN score was originally developed to measure the accuracy of written health information. The questionnaire has been found effective in analyzing the quality of health information on the Internet. Ferhatoglu and colleagues evaluated the quality of YouTube videos and obesity surgery regarding uploading sources, and the authors found a significantly higher DISCERN score for videos shared by professional health providers [12]. In accordance, Yuksel and Cakmak achieved statistically better DISCERN scores for YouTube videos on COVID-19 and pregnancy uploaded by health workers [13]. Accordingly, informative videos in the present study had a significantly better DISCERN score than patients' experience videos and new agency videos.

Nagpal and colleagues developed the MICI score to evaluate the quality of videos about the Ebola pandemic [14]. Previous reports had contradictory results regarding informative videos' MICI scores about COVID-19. Ho et al. found similar MICI scores among COVID-19 videos from different sources, including government agencies, independent users, and news agencies in the Korean language [15]. In the study by Atac's team, which investigated the video quality of COVID-19 and general public health by using the MICI score, the MICI score was 2.76 for videos in English and 3.33 for videos in Turkish [16]. Dutta et al. analyzed the videos about COVID-19 in six different languages (Arabic, Bengali, Dutch, English, Hindi, and Nigerian), and the mean score of MICI was 5.68 [17]. In the present study, the MICI score was 13.0 in videos about COVID-19 and gynecologic cancers. Compared to other studies, the period between the COVID-19 pandemic announcement and current study conduct is more extended. Thus, video uploaders may have more knowledge about COVID-19, which may have played a role in higher MICI scores in the present study.

The Video Information and Quality Index are defined to analyze the overall video quality. Ozdede and Peker investigated the quality of YouTube videos about COVID-19 and dentistry; the authors claimed that helpful videos had a significantly higher VIQI score [18]. In another study, Hatipoglu and Gas examined the quality of YouTube videos about surgically assisted rapid palatal enlargement. The authors state that videos with moderate content had significantly higher VIQI scores when compared to low-content videos [19]. We analyzed the videos about COVID-19 and gynecologic cancers for the first time, and we found a significantly higher total score of VIQI in informative videos.

Previous studies that investigated YouTube videos about different diseases had dissimilar results regarding the number of views and 'dislikes' [20]. In this particular study, we found a significantly higher view number in patients' experience videos. We believe two reasons may have played a role in this result. Firstly, people like to empathize with patients and listen to patient stories. Secondly, the most significant news information sources are televisions in our country. It explains the lower view rate of news agency videos. Also, the informative rate had a significantly lower dislike rate. We can indirectly infer that people like to receive information from professional sources.

Although this is the first study to analyze the characteristics of YouTube videos about COVID-19 and gynecologic cancers, the present study has some limitations. We only chose YouTube videos in the Turkish language, and we did not compare these videos with any videos in other languages. Secondly, the first COVID-19 case was detected on March 11, 2020. Thus, we evaluated videos of almost eight months duration. Also, knowledge about COVID-19 is increasing rapidly. Hence, the contents of videos are changing during the pandemic. The video quality of uploaded YouTube videos may be the subject of another study. Moreover, we searched the five most common term groups on YouTube, expanding keywords during the search associated with COVID-19 and gynecological cancer to increase the number of videos.

## Conclusions

In conclusion, YouTube videos about COVID-19 and gynecological cancers are easily accessible resources. This study shows that the videos in which patients share their experiences about their cancers are watched considerably more, and the more informative videos have fewer dislike clicks. Also, informative videos have acceptable quality, according to the MICI score. Additionally, informative videos had considerably better DISCERN and VIQI scores than patient-produced videos, which included the self-experiences of patients and videos of news agencies. Overall, with an improvement in video quality, YouTube videos could become a reference source with possible impacts of COVID-19 on the awareness of Turkish people about gynecologic cancers.
